# Impact of prolonged postoperative antibiotic administration on postoperative complications in elderly patients undergoing colorectal cancer surgery

**DOI:** 10.3389/fsurg.2025.1734652

**Published:** 2026-01-12

**Authors:** Go Woon Park, Nahyeon Park, Jung Chul Kuk, Dae Ro Lim, Eung Jin Shin

**Affiliations:** Division of Colon and Rectal Surgery, Department of Surgery, Soonchunhyang University College of Medicine, Soonchunhyang University Bucheon Hospital, Bucheon, Republic of Korea

**Keywords:** colorectal cancer, postoperative complication, prolonged antibiotic usage, surgery, the elderly

## Abstract

**Propose:**

The purpose of this study was to investigate whether prolonged antibiotic usage beyond conventional practices could impact the incidence of postoperative complications in elderly patients undergoing colorectal cancer surgery.

**Materials and methods:**

Between January 2016 and December 2022, a total of 292 patients aged over 70 years who underwent curative resection for colorectal cancer were identified from a retrospective database. The study population was divided into two groups: the POD#1 group, consisting of patients who received postoperative antibiotics for 1 day (*n* = 214), and the POD#3 group, comprising patients who received postoperative antibiotics for at least 3 days (*n* = 78).

**Results:**

A significant difference between two groups was observed in the rates of total postoperative complications, infection-related complications and antibiotic reuse. In the POD#1 group, complications occurred at a rate of 25.7% (*n* = 55), while the POD#3 group showed a lower complication rate of 14.1% (*n* = 11) (*p* = 0.036). The infection-related complications such as anastomotic leakage, wound infection, pneumonia occurred at a rate of 20.1% (*n* = 43) in POD#1 group compared to 5.1% (*n* = 4) in POD#3 group (*p* = 0.002). The antibiotic re-use rate was 22.4% (*n* = 48) in the POD#1 group and 11.5% (*n* = 9) in the POD#3 group (*p* = 0.038). In the multivariate analysis, ASA score (HR 1.79; 95% CI 1.09–2.97; *p*-value = 0.022) and postoperative antibiotic usage (HR 0.49; 95% CI 0.26–0.94; *p*-value = 0.032) emerged as independent risk factors for postoperative complications.

**Conclusion:**

In colorectal cancer patients over the age of 70, prolonged antibiotic usage demonstrated a reduction in the rate of postoperative complications, including anastomotic leakage. Considering the potentially fatal consequences of postoperative complications in the elderly, the advantages of extended antibiotic administration beyond conventional usage outweigh the concerns regarding complications or costs associated with antibiotics use.

## Introduction

Improvements in healthcare services and living conditions are leading to a rapid increase in the elderly population worldwide. As life expectancy increases, the incidence of colorectal cancer in the patients of advanced age is also increasing, leading to a rise in the number of older adults requiring surgical treatments (1). Because surgery is the only curative treatment for colorectal cancer, even in the elderly patient, surgical resection is being actively performed (2). As the advanced age population is expected to grow continuously, and age is a significant risk factor for colorectal cancer, the diagnosis and treatment of colorectal cancer in this age are anticipated to further increase (3).

While the health of the older adults has improved significantly, it is essential to acknowledge that despite these advancements, age-related physiological changes lead to a reduced capacity to tolerate stress. Factors such as diminished immune function and impaired tissue healing contribute to this vulnerability compared to younger individuals (4). Elderly patients commonly present with multiple comorbidities including hypertension, diabetes, cardiovascular disease, pulmonary disease, and renal dysfunction, which are more common in this age group and contribute to increased surgical risk. Hence, these patients frequently face elevated postoperative risks and have a higher susceptibility to infectious complications, which can lead to potentially life-threatening outcomes (5).

In recent years, there has been a growing body of research exploring the optimal strategies for minimizing postoperative complications in elderly patients. By extending the duration of postoperative antibiotic prophylaxis in elderly patients, who appears to have the potential to reduce postoperative complications. This study aimed to evaluate whether prolonged postoperative antibiotics administration is associated with improved postoperative complications in elderly (≥70 aged) patients undergoing curative colorectal cancer surgery.

## Materials and methods

### Study design and setting

This study was designed as a retrospective cohort study conducted at Soonchunhayng University Bucheon Hospital, a tertiary referral center in South Korea. Clinical, operative, and pathological data were extracted from the institutional electronic medical record (EMR)-based colorectal surgery database. All data were reviewed for patients treated between January 2016 and December 2022, with follow-up continued until December 2023. This retrospective cohort study was conducted and reported in accordance with the STROBE guidelines for observational studies.

### Patients

Between January 2016 and December 2022, a total of 292 patients aged 70 years or older who underwent curative resection for colorectal cancer were identified from a retrospective database. Inclusion criteria were: (1) age ≥70 years, (2) histologically confirmed colorectal adenocarcinoma, and (3) curative-intent surgical resection. Exclusion criteria included: (1) stage IV disease, (2) synchronous or metachronous colorectal cancer, (3) palliative surgery or bypass procedures, and (4) incomplete medical records. The study was divided into two groups and analyzed. A total of 292 patients met the inclusion criteria. All surgery was performed elective surgery. Patients were categorized based on postoperative antibiotic duration into two group:

- POD#1 group: postoperative antibiotics discontinued within postoperative day 1 (*n* = 214)
- POD#3 group: postoperative antibiotics continued for ≥3 postoperative days (*n* = 78)

In the POD#3 group, considering the median range of the group that continuously used antibiotics, we categorized it as 3 days or more. Prolongation beyond postoperative day 3 was mainly based on clinical judgement, including higher ASA score, presence of multiple comorbidities, suspected intraoperative contamination, or early postoperative signs suggestive of increased infection risk. All data on clinical and pathological features were reviewed retrospectively. All patients underwent colonoscopy, biopsy, and staging scans [chest, abdomen, and pelvis computed tomography (CT) scans], and occasionally, positron emission tomography scans were performed. All patients underwent curative resection. Rectal cancer patients with clinically T3 or T4 and/or node positive received long-course neoadjuvant chemoradiation therapy (CRT) (five cycles of fluorouracil based chemotherapy and 50.4 Gy). Patients received close follow-up and were included in a database until December 2023 or their death if this occurred before December 2023.

### Exposure and outcome definitions

#### Exposure (independent variable)

- Duration of postoperative systemic antibiotic administration (1 vs. ≥3 days)
- Antibiotic regimen followed institutional colorectal surgery protocol (2nd generation cephalosporin or 3rd-generation cephalosporin ± metronidazole, or piperacillin-tazobactam)

All patients received a standard preoperative prophylactic regimen consisting of 2nd generation cephalosporin administered within 60 min prior to skin incision, according to institutional protocol. Postoperative antibiotic regimen were 2nd generation cephalosporin or 3rd-generation cephalosporin ± metronidazole, or piperacillin-tazobactam.

#### Primary outcome

- Total postoperative complications, defined as any complication occurring within 30 postoperative days.

#### Secondary outcomes

- Infection-related complications, including;
- Anastomotic leakage (clinical or radiologic confirmation),
- Surgical site infection (CDC criteria),
- Pneumonia (clinical symptoms + radiologic evidence),
- Intra-abdominal abscess,
- Colitis,
- Urinary tract infection (urinalysis ± cuture).

### Bias and confounding control

To reduce selection bias, uniform inclusion/exclusion criteria were applied. To minimize information bias, all complications were adjudicated independently by two colorectal surgeons. Potential confounders (age, sex, BMI, ASA score, tumor stage, surgical method) were adjusted for using multivariate logistic regression. No imputation was required as missing data were minimal (<1%) and managed by complete-case analysis.

### Statistical analysis

Categorical variables were analyzed using the *χ*^2^ or Fisher's exact tests. Continuous variables were compared using Student's *t* test or Mann–Whitney *U* rank test, as appropriate. Variables with *p* < 0.10 in univariate analysis or recognized as clinically relevant were entered into the multivariate logistic regression model to identify independent risk factors for postoperative complications. Result were reported as hazard ratios (HRs) with 95% confidence intervals (CIs). All statistical analyses were performed using SAS Version 9.1.3 (SAS Institute Inc., Cary, NC) and SPSS Version 24.0. A two-sided *p*-value <0.05 was considered statistically significant.

### Ethics

This study was approved by the institutional Review Board of Soonchunhyang University Bucheon Hospital (IRB No: SCHBC2025-05-012). Given the retrospective study design and anonymized data collection, the requirement for informed consent was waived.

## Results

### Patients characteristics

A total of 292 patients were included in the analysis, with 214 receiving antibiotics postoperatively 1 day (POD#1 group) and 78 receiving antibiotics for at least 3 days postoperatively (POD#3 group) (Table 1). Mean age, sex ratio, weight, height and body mass index (BMI) have no significant difference between the two groups. Laparoscopic or robotic surgery were performed 93.0% (*n* = 199) in POD#1 group and 88.5% (*n* = 69) in POD#3 group. There was no significant difference in the location of the lesion between the two groups.

**Table 1:** Patient characteristics (*n* = 292).

	POD#1 group (*n* = 214) (%)	POD#3 group (*n* = 78) (%)	*p* value
Age [mean ± SD, (range)] (year)	76.4 ± 4.5 (70–92)	77.7 ± 4.7 (70–88)	0.500
Sex, *n* (%)			0.903
Male	95 (44.4%)	34 (43.6%)	
Female	119 (55.6%)	44 (56.4%)	
Weight [mean ± SD, (range)] (kg)	59.2 ± 10.0 (33.3–92.0)	57.4 ± 10.6 (32.0–83.0)	0.649
Height [mean ± SD, (range)] (cm)	158.0 ± 8.6 (133.3–174.0)	156.5 ± 8.6 (135.5–175.0)	0.965
BMI [mean ± SD, (range)] (kg/m^2^)	23.7 ± 3.6 (13.9–38.4)	23.4 ± 3.8 (14.6–33.1)	0.414
ASA score, *n* (%)			0.206
1	19 (8.9%)	2 (2.6%)	
2	141 (65.9%)	50 (64.1%)	
3	52 (24.3%)	25 (32.0%)	
4	2 (0.9%)	1 (1.3%)	
Surgical method			0.212
Open	15 (7.0%)	9 (11.5%)	
Laparoscopic or robotic	199 (93.0%)	69 (88.5%)	
Lesion location			0.251
Right side	61 (28.5%)	27 (34.6%)	
Left side	103 (48.1%)	29 (37.2%)	
Rectum	50 (23.4%)	22 (28.2%)	

### Pathologic results

Colorectal cancer are classified according to the 8th edition of American Joint committees on Cancer (AJCC) (Table 2). In the POD#1 group, stage III was the most common at 34.6%, followed by stage II at 33.6%, and then stage I at 26.6%. Conversely, in the POD#3 group, stage 2 was the most prevalent at 42.3%, followed by stage 3 at 35.9%, and stage I at 19.2%. However, no statistically significant difference was observed between the two groups. In both groups, the majority exhibited a moderate degree of differentiation (78.0% vs. 78.2%). The mean number of harvested lymph nodes was 20.8 and 25.9, respectively, with no significant difference observed. The presence of lymphovascular invasion was 83.2% in POD#1 group and 78.2% in POD#3 group. The perineural invasion was 58.4% vs. 39.7% in each groups respectively. The proximal resection margin and distal resection margin measured 9.3 and 9.7 cm, and 6.3 and 7.1 cm, respectively, with no statistically significant difference.

**Table 2:** Postoperative pathologic outcomes (*n* = 292).

	POD#1 group (*n* = 214) (%)	POD#3 group (*n* = 78) (%)	*p* value
pTNM stage, no. (%)			0.341
0	11 (5.1%)	2 (2.6%)	
I	57 (26.6%)	15 (19.2%)	
II	72 (33.6%)	33 (42.3%)	
III	74 (34.6%)	28 (35.9%)	
pT stage, no. (%)			0.000
0	12 (5.6%)	2 (2.6%)	
1	32 (14.9%)	6 (7.7%)	
2	36 (16.8%)	9 (11.5%)	
3	123 (57.5%)	42 (53.8%)	
4	11 (5.1%)	19 (24.4%)	
pN stage, no. (%)			0.215
0	140 (65.4%)	50 (64.1%)	
1	53 (24.8%)	15 (19.2%)	
2	21 (9.8%)	13 (16.7%)	
Grade of differentiation, no. (%)			0.074
Well	29 (13.6%)	5 (6.4%)	
Moderate	167 (78.0%)	61 (78.2%)	
Poor	15 (7.0%)	8 (10.2%)	
Mucinous	3 (1.4%)	4 (5.1%)	
Harvested no. of lymph nodes, (mean ± SD), (no)	20.8 ± 11.8	25.9 ± 12.3	0.265
Lymphovascular invasion			0.329
No	178 (83.2%)	61 (78.2%)	
Yes	36 (16.8%)	17 (21.8%)	0.005
Perineural invasion			
No	125 (58.4%)	31 (39.7%)	
Yes	89 (41.6%)	47 (60.3%)	
PRM, (mean ± SD), (cm)	9.3 ± 5.9	9.7 ± 6.5	0.333
DRM, (mean ± SD), (cm)	6.3 ± 6.0	7.1 ± 8.3	0.094

### Postoperative outcomes

**Figure 1:**
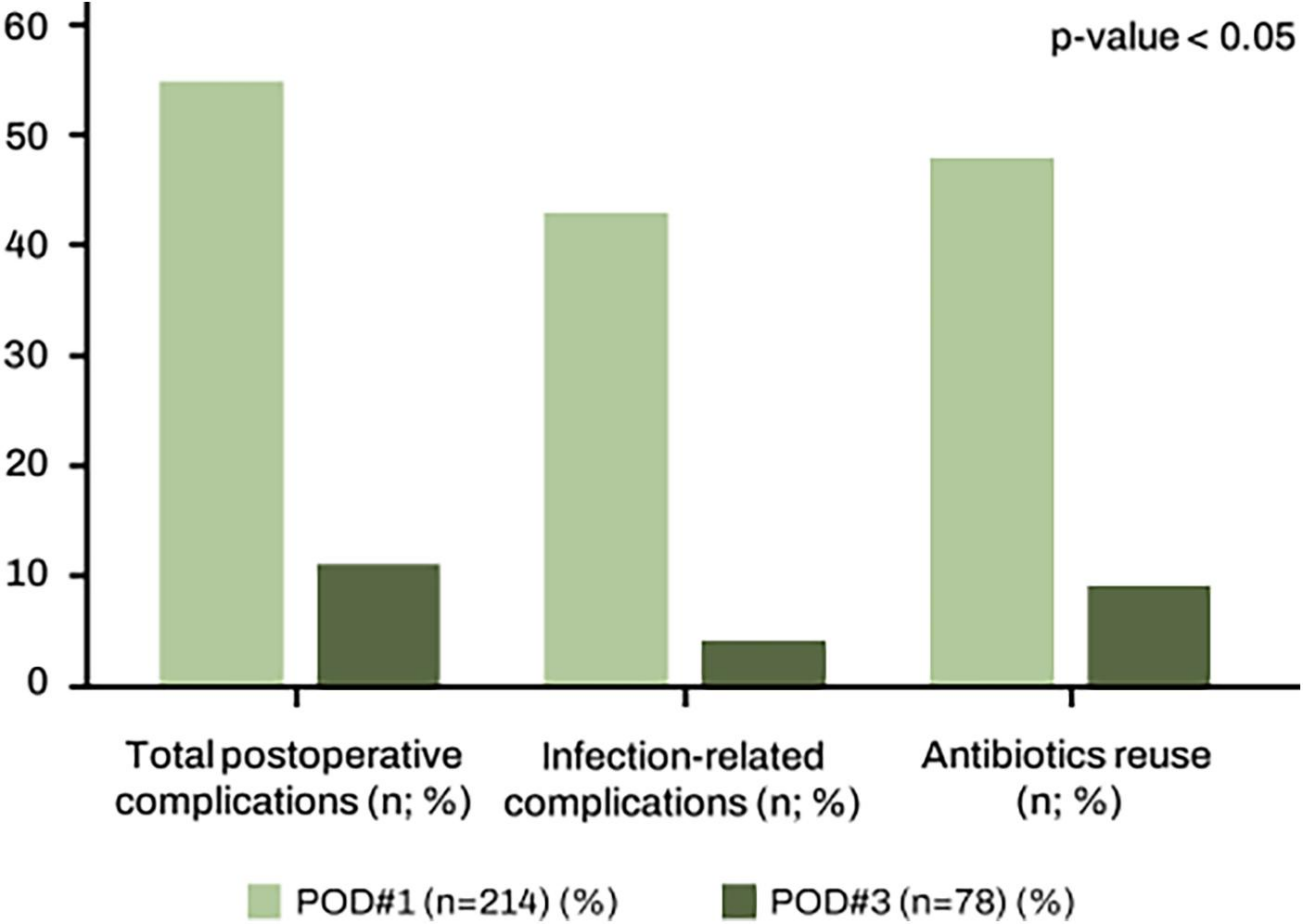
A significant difference between two groups was observed in the rates of total postoperative complications, infection-related complications and antibiotic reuse.

The postoperative outcomes are described in Table 3. A significant difference between two groups was observed in the rates of total postoperative complications, infection-related complications and antibiotic re-use (Figure 1). In the POD#1 group, complications occurred at a rate of 25.7% (*n* = 55), while the POD#3 group showed a lower complication rate of 14.1% (*n* = 11) (*p* = 0.036). The infection-related complications such as anastomotic leakage, wound infection, pneumonia occurred at a rate of 20.1% (*n* = 43) in POD#1 group compared to 5.1% (*n* = 4) in POD#3 group (*p* = 0.002). When classified according to the Clavien-Dindo system, the POD#3 group showed a lower incidence of grade (I–II) complication compared with the POD#1 group (11.5% vs. 22.5%, *p* < 0.05) while high grade (III–IV) complications were similar between group. The patients in the POD#1 group to whom antibiotics reintroduced during hospitalization due to necessity, and the patients in the POD#3 group to whom antibiotics were discontinued and then resumed were categorized as antibiotic reutilization. The antibiotic reuse rate was 22.4% (*n* = 48) in the POD#1 group and 11.5% (*n* = 9) in the POD#3 group (*p* = 0.038). Mortality occurred exclusively in the group that used antibiotics perioperatively, and the causes of death were pelvic sepsis due to anastomotic leakage and pneumonia. The 30-day readmission rate was 5.1% (*n* = 11) in the POD#1 group and 3.8% (*n* = 3) in the POD#3 group (*p* = 0.647). The emergency room visit rates within 30 days was 6.5% (*n* = 14) in the POD#1 group and 3.8% (*n* = 3) in the POD#3 group (*p* = 0.384), but there was no statistically significant difference. The average length of hospital stay was also comparable between the two groups, at 10.5 and 10.8 days, with no significant difference.

**Table 3:** Postoperative outcomes (*n* = 292).

	POD#1 group (*n* = 214) (%)	POD#3 group (*n* = 78) (%)	*p* value
Total postoperative complications (*n*; %)	55 (25.7%)	12 (15.4%)	0.036
Infection related complications (*n*; %)	43 (20.1%)	4 (5.1%)	0.002
Anastomosis site leakage	9 (4.2%)	1 (1.3%)	
Wound infection	13 (6.1%)	2 (2.6%)	
Pneumonia	12 (5.6%)	0 (0.0%)	
Intraabdominal abscess	2 (0.9%)	1 (1.3%)	
Colitis	5 (2.3%)	0 (0.0%)	
Acute pyelonephritis	1 (0.5%)	1 (1.3%)	
Phlebitis	1 (0.5%)	0 (0.0%)	
Other complications (*n*; %)	19 (8.9%)	8 (10.3%)	0.495
Clavien–Dindo grades			
I–II	48 (22.4%)	9 (11.5%)	0.042
III–IV	7 (3.3%)	3 (3.8%)	0.376
Antibiotics reuse (*n*; %)	48 (22.4%)	9 (11.5%)	0.038
Total Mortality	2 (0.9%)	0 (0.0%)	0.392
Readmission within 30 days (*n*; %)	11 (5.1%)	3 (3.8%)	0.647
Visit emergency within 30 days (*n*; %)	14 (6.5%)	3 (3.8%)	0.384
Hospital stay [mean ± SD, (range)] (day)	10.5 ± 5.8 (7–44)	10.8 ± 3.8 (7–23)	0.217

### Univariate and multivariate analysis for risk factor for postoperative complications

The results of univariate and multivariate analyses are summarized in Table 4. Univariate analysis demonstrates a significant relationship between postoperative complications and sex, ASA score, and postoperative antibiotic usage. In the multivariate analysis, ASA score (HR 1.79; 95% CI 1.09–2.97; *p*-value = 0.022) and postoperative antibiotic usage (HR 0.49; 95% CI 0.26–0.94; *p*-value = 0.032) emerged as independent risk factors for postoperative complications.

**Table 4:** Univariate and multivariate analysis for risk factor of postoperative complication.

Factors	Univariate analysis			Multivariate analysis		
	HR	95% CI	*p*-value	HR	95% CI	*p*-value
Age, years (70–79 vs. ≥80)	0.69	0.38–1.22	0.200			
Sex (male vs. female)	0.57	0.34–0.96	0.031	0.61	0.63–1.03	0.065
BMI (<25 vs. ≥25)	0.97	0.58–1.61	0.897			
ASA score (≤2 vs. >2)	1.82	1.12–3.00	0.016	1.79	1.09–2.97	0.022
Stage (I–II vs. III)	1.16	0.71–1.91	0.554			
T stage (0–2 vs. 3–4)	1.06	0.63–1.77	0.833			
Surgical type (open vs. minimal invasive)	0.61	0.29–1.28	0.195			
Tumor location (right side vs. left side)	1.16	0.84–1.61	0.377			
Tumor location (colon vs. rectum)	0.17	0.86–2.41	0.168			
Postoperative prophylactic antibiotics (≤1 vs. ≥3 days)	0.52	0.27–1.00	0.047	0.49	0.26–0.94	0.032

## Discussion

**Figure 2:**
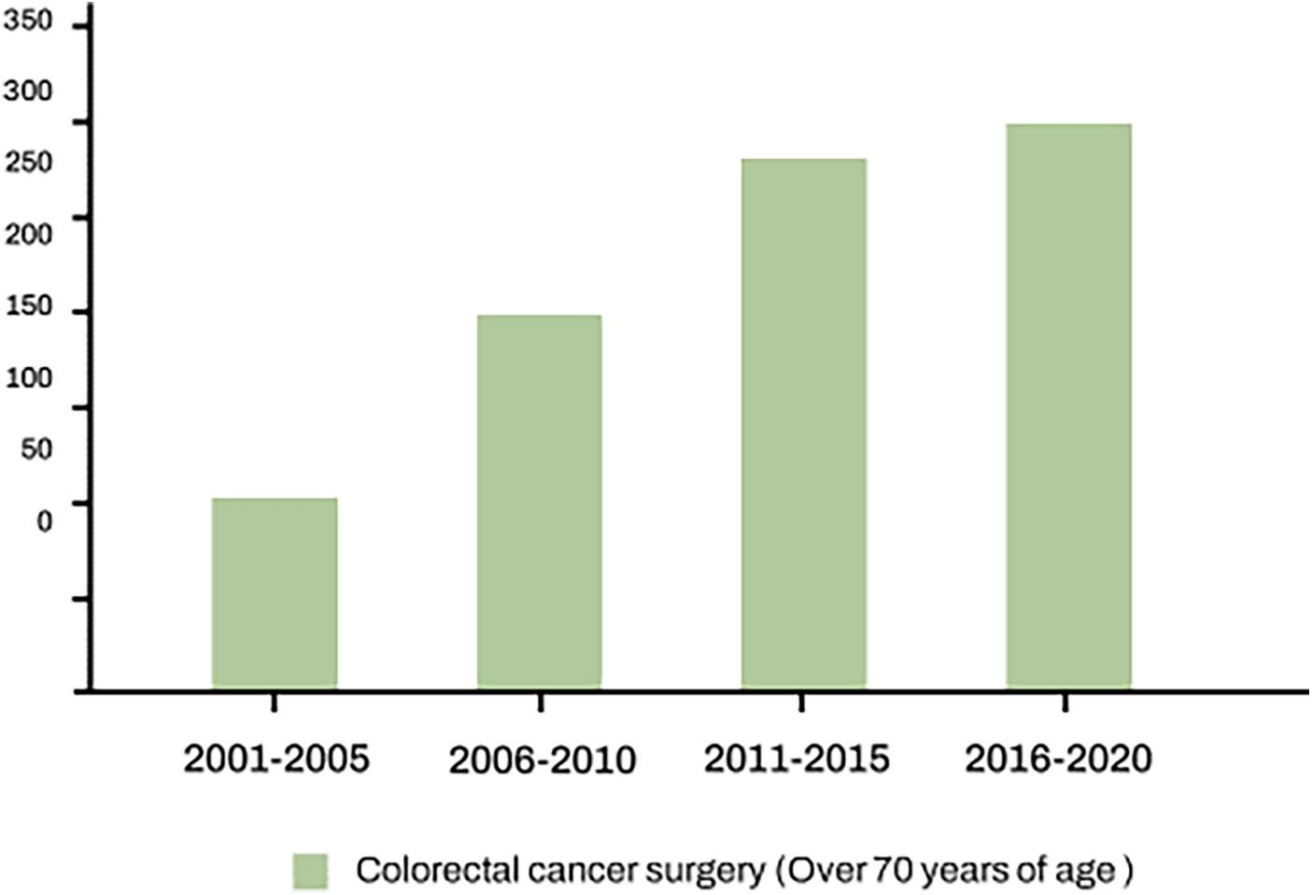
Number of colorectal cancer surgery with over 70 years of age in present institution. It shows that the number of surgeries for colorectal cancer in elderly is gradually increasing.

According to the World Health Organization GLOBOCAN database, colorectal cancer ranks as the third most commonly diagnosed cancer in males and the second most commonly diagnosed in females globally, with expectations of continued increase (6). Similarly, at the present institution, the number of colorectal cancer surgeries in patients over 70 years of age is gradually increasing (Figure 2). Elderly patients exhibited a heightened incidence of postoperative complications, notably infection-related issues including surgical site infections, urinary tract infections, and respiratory infections (7, 8). As a result, this leads to an increase in both the length of hospital stay and admission rate to intensive care units, along with higher rates of morbidity and mortality (9, 10). While complication prevention is important for all age groups, tailored strategies for complication prevention are necessary considering the specificity of the elderly population (11–13). Recently, in colorectal surgery, the combination of oral antibiotics with mechanical bowel preparation is recommended, given its advantages in terms of reducing anastomotic leakage, morbidity, mortality, and healthcare costs (14). Conversely, it has been established that the routine use of antibiotics for postoperative days does not provide any additional benefits (15). However, these studies were conducted on relatively younger populations, and the benefits of postoperative antibiotic use in the elderly groups have not been definitively established.

Anastomotic leakage after colorectal surgery is devastating complication resulting in significantly increased morbidity, mortality, and medical expense. The rate of anastomotic leakage, approximately 10 percent, varies based on the location of the anastomosis and differs among studies due to variations in its definition (16, 17). The human intestinal microbiome is believed to intricately contribute to the pathogenesis of physical conditions such as obesity, gastrointestinal malignancies, and Crohn's disease (18, 19). However, its impact on various diseases remains largely undiscovered. The healing and disruption of anastomosis after colorectal resection is one of the relatively unexplored area where intraluminal microbes are anticipated to play a crucial role.

The first investigation that evaluates effect of the microbiome on colonic anastomosis administered antibiotics directly to the anastomotic site via an indwelling catheter (20). The study group demonstrated complete anastomotic healing, while the control group which administered saline without antibiotics developed leakage, peritonitis, and death. Another similar study in the rats exhibited the healing effect of the non-absorbable oral antibiotics on colonic anastomosis (21). In applying these findings to a prospective, randomized, double-blind and placebo-controlled clinical multicenter trial, the patients with oral decontamination, from preoperative day to postoperative day 7, after total gastrectomy for gastric cancer showed lower mortality and fewer anastomotic leakage compared to the placebo group (22–24). The surgical group applied this protocol to colorectal surgery (25). The treatment group was administered oral antibiotics starting the day before surgery until postoperative day 7, demonstrating a lower rate of anastomotic leakage and infectious complications compared to the placebo group. In present study, the patients were administered systemic antibiotics instead of oral antibiotics and this is significant because, while the incidence rate of anastomotic leakage is generally known to be around 10%, in this study, the POD#1 group exhibited a lower incidence rate of 4.2%, and the POD#3 group showed an even lower rate of 1.3%.

All surgeries conducted under general anesthesia carry the risk of developing postoperative pneumonia, which can lead to substantial morbidity and mortality. The incidence of postoperative pneumonia has been reported to range from 2.2% to 13% (26), but more prevalent in the elderly patients with old age itself being a risk factor (27). General anesthesia and surgery, along with consequent atelectasis, alter lung mechanics and breathing patterns, thereby making the lung defense system more susceptible to infection (28, 29). Additionally, the incidence of postoperative pneumonia tends to be higher in patients with atelectasis, which occurs more frequently in old ages (30–32). Although the use of postoperative antibiotics to prevent or reduce the incidence of postoperative pneumonia haven't been established, this study is significant in that the incidence of postoperative pneumonia in the POD#3 group was 0% compared to 5.6% in the POD#1 group. Given that postoperative pneumonia is more fatal in the elderly, it is necessary to consider additional antibiotic use beyond conventional practice.

In this retrospective cohort study of 292 patients aged ≥70 years undergoing curative colorectal cancer surgery, prolonged postoperative antibiotic administration (≥3 days) was associated with a significantly lower rate of overall complications, infection-related complications, and postoperative antibiotic reuse compared with standard discontinuation on postoperative day 1. Notably, the POD#3 group demonstrated markedly reduced rates of anastomotic leakage and pneumonia, both of which are particularly detrimental in elderly and comorbid populations. Although our results differ from guideline recommendations, they should not be interpreted as evidence to change clinical practice. Instead, they highlight a potential gap in current evidence: the lack of prospective studies focusing on high-risk elderly surgical patients. The reduced rates of anastomotic leakage and pneumonia observed in our study may warrant further evaluation in controlled trials targeting this vulnerable population.

This study has several limitations. Its retrospective design introduces the potential for selection bias, and decisions regarding prolonged antibiotics were at the discretion of treating surgeons. Residual confounding may remain despite multivariate adjustment. Additionally, this is a single-center experience and may not be generalizable to other institutions with different perioperative protocols. Nevertheless, the consistent benefit observed across multiple clinically relevant outcomes suggests that postoperative antibiotic duration may merit further investigation in elderly colorectal cancer patients.

## Conclusion

In conclusion, while prophylactic guidelines discourage extended postoperative antibiotics for the general population, the present study data indicate a possible role for selective use in order, high risk patients. Prospective randomized studies are needed to validate these findings and to better defined which elderly patients may benefit from prolonged postoperative prophylaxis.

## Data Availability

The datasets presented in this article are not readily available because this study is a medical chart review study. Requests to access the datasets should be directed to Daero Lim, limdaero@schmc.ac.kr.

## References

[1] Watters JM. Surgery in the elderly. Can J Surg. (2002) 45(2):104. 11939651 PMC3686930

[2] Lemmens VE Janssen-Heijnen ML Houterman S Verheij KD Martijn H van de Poll-Franse L Which comorbid conditions predict complications after surgery for colorectal cancer? World J Surg. (2007) 31(1):192–9. 10.1007/s00268-005-0711-8 17180570

[3] Rawla P Sunkara T Barsouk A. Epidemiology of colorectal cancer: incidence, mortality, survival, and risk factors. Prz Gastroenterol. (2019) 14(2):89–103. 10.5114/pg.2018.81072 31616522 PMC6791134

[4] Evers BM Townsend CM Jr Thompson JC. Organ physiology of aging. Surg Clin North Am. (1994) 74(1):23–39. 10.1016/S0039-6109(16)46226-2 8108769

[5] Seymour DG Pringle R. Post-operative complications in the elderly surgical patient. Gerontology. (1983) 29(4):262–70. 10.1159/000213125 6873640

[6] Morgan E Arnold M Gini A Lorenzoni V Cabasag CJ Laversanne M Global burden of colorectal cancer in 2020 and 2040: incidence and mortality estimates from GLOBOCAN. Gut. (2023) 72(2):338–44. 10.1136/gutjnl-2022-327736 36604116

[7] Frago R Ramirez E Millan M Kreisler E del Valle E Biondo S. Current management of acute malignant large bowel obstruction: a systematic review. Am J Surg. (2014) 207(1):127–38. 10.1016/j.amjsurg.2013.07.027 24124659

[8] Grosso G Biondi A Marventano S Mistretta A Calabrese G Basile F. Major postoperative complications and survival for colon cancer elderly patients. BMC Surg. (2012) 12:1–5. 10.1186/1471-2482-12-S1-S20 23173563 PMC3499273

[9] Colorectal Canc Collaborative Group, Best L Baughan C Buchanan R Davis C Fentiman I Surgery for colorectal cancer in elderly patients: a systematic review. Lancet. (2000) 356(9234):968–74. 10.1016/S0140-6736(00)02713-6 11041397

[10] Tahiri M Sikder T Maimon G Teasdale D Hamadani F Sourial N The impact of postoperative complications on the recovery of elderly surgical patients. Surg Endosc. (2016) 30:1762–70. 10.1007/s00464-015-4440-2 26194260

[11] Bernard H Cole W. The prophylaxis of surgical infection: the effect of prophylactic antimicrobial drugs on the incidence of infection following potentially contaminated operations. Surgery. (1964) 56:151–7. 14174732

[12] Miles A Miles EM Burke J. The value and duration of defence reactions of the skin to the primary lodgement of bacteria. Br J Exp Pathol. (1957) 38(1):79. 13413084 PMC2082171

[13] Polk HC Jr Christmas AB. Prophylactic antibiotics in surgery and surgical wound infections. Am Surg. (2000) 66(2):105–11. 10.1177/000313480006600203 10695738

[14] Migaly J Bafford AC Francone TD Gaertner WB Eskicioglu C Bordeianou L The American society of colon and rectal surgeons clinical practice guidelines for the use of bowel preparation in elective colon and rectal surgery. Dis Colon Rectum. (2019) 62(1):3–8. 10.1097/DCR.0000000000001238 30531263

[15] Stone HH Haney BB Kolb LD Geheber CE Hooper CA. Prophylactic and preventive antibiotic therapy: timing, duration and economics. Ann Surg. (1979) 189(6):691–9. 10.1097/00000658-197906000-00004 378140 PMC1397224

[16] Branagan G Finnis D. Prognosis after anastomotic leakage in colorectal surgery. Dis Colon Rectum. (2005) 48:1021–6. 10.1007/s10350-004-0869-4 15789125

[17] Shogan BD Carlisle EM Alverdy JC Umanskiy K. Do we really know why colorectal anastomoses leak? J Gastrointest Surg. (2013) 17(9):1698–707. 10.1007/s11605-013-2227-0 23690209

[18] Paun BC Cassie S MacLean AR Dixon E Buie WD. Postoperative complications following surgery for rectal cancer. Ann Surg. (2010) 251(5):807–18. 10.1097/SLA.0b013e3181dae4ed 20395841

[19] Morowitz MJ Babrowski T Carlisle EM Olivas A Romanowski KS Seal JB The human microbiome and surgical disease. Ann Surg. (2011) 253(6):1094–101. 10.1097/SLA.0b013e31821175d7 21422915 PMC4854204

[20] Cohn I Jr Rives JD. Antibiotic protection of colon anastomoses. Ann Surg. (1955) 141(5):707. 10.1097/00000658-195505000-00016 14362409 PMC1609892

[21] Cohen S Cornell C Collins M Sell J Blanc W Altman R. Healing of ischemic colonic anastomoses in the rat: role of antibiotic preparation. Surgery. (1985) 97(4):443–6. 3983820

[22] Schardey H Kamps T Rau H Gatermann S Baretton G Schildberg F. Bacteria: a major pathogenic factor for anastomotic insufficiency. Antimicrob Agents Chemother. (1994) 38(11):2564–7. 10.1128/AAC.38.11.2564 7872748 PMC188242

[23] Schardey HM Joosten U Finke U Staubach KH Schauer R Heiss A The prevention of anastomotic leakage after total gastrectomy with local decontamination: a prospective, randomized, double-blind, placebo-controlled multicenter trial. Ann Surg. (1997) 225(2):172–80. 10.1097/00000658-199702000-00005 9065294 PMC1190646

[24] Gong W Li J. Combat with esophagojejunal anastomotic leakage after total gastrectomy for gastric cancer: a critical review of the literature. Int J Surg. (2017) 47:18–24. 10.1016/j.ijsu.2017.09.019 28935529

[25] Schardey H Wirth U Strauss T Kasparek M Schneider D Jauch K. Prevention of anastomotic leak in rectal cancer surgery with local antibiotic decontamination: a prospective, randomized, double-blind, placebo-controlled single center trial. Int J Colorectal Dis. (2020) 35:847–57. 10.1007/s00384-020-03544-8 32103326

[26] Chughtai M Gwam CU Mohamed N Khlopas A Newman JM Khan R The epidemiology and risk factors for postoperative pneumonia. J Clin Med Res. (2017) 9(6):466–75. 10.14740/jocmr3002w 28496546 PMC5412519

[27] Xiang B Jiao S Yuan F. Risk factors for postoperative pneumonia: a case-control study. Front Public Health. (2022) 10:913897. 10.3389/fpubh.2022.913897 35875004 PMC9304902

[28] Duggan M Kavanagh BP Warltier DC. Pulmonary atelectasis: a pathogenic perioperative entity. Anesthesiology. (2005) 102(4):838–54. 10.1097/00000542-200504000-00021 15791115

[29] Ko E Yoo KY Lim CH Jun S Lee K Kim YH. Is atelectasis related to the development of postoperative pneumonia? A retrospective single center study. BMC Anesthesiol. (2023) 23(1):77. 10.1186/s12871-023-02020-4 36906539 PMC10007747

[30] Cassidy MR Rosenkranz P McCabe K Rosen JE McAneny D. I COUGH: reducing postoperative pulmonary complications with a multidisciplinary patient care program. JAMA Surg. (2013) 148(8):740–5. 10.1001/jamasurg.2013.358 23740240

[31] Roberts RR Hota B Ahmad I Scott RD Foster SD Abbasi F Hospital and societal costs of antimicrobial-resistant infections in a Chicago teaching hospital: implications for antibiotic stewardship. Clin Infect Dis. (2009) 49(8):1175–84. 10.1086/605630 19739972

[32] Daneman N Gruneir A Bronskill SE Newman A Fischer HD Rochon PA Prolonged antibiotic treatment in long-term care: role of the prescriber. JAMA Intern Med. (2013) 173(8):673–82. 10.1001/jamainternmed.2013.3029 23552741

